# Phosphoproteomic analysis of *Methanohalophilus portucalensis* FDF1^T^ identified the role of protein phosphorylation in methanogenesis and osmoregulation

**DOI:** 10.1038/srep29013

**Published:** 2016-06-30

**Authors:** Wan-Ling Wu, Shu-Jung Lai, Jhih-Tian Yang, Jeffy Chern, Suh-Yuen Liang, Chi-Chi Chou, Chih-Horng Kuo, Mei-Chin Lai, Shih-Hsiung Wu

**Affiliations:** 1 Institute of Biological Chemistry, Academia Sinica, Taipei 11529, Taiwan; 2 Department of Life Sciences, National Chung Hsing University, Taichung 40227, Taiwan; 3 Ph.D program in Microbial Genomics, National Chung Hsing University and Academia Sinica, Taichung 40227, Taiwan; 4 Chemical Biology and Molecular Biophysics Program, Taiwan International Graduate Program, Academia Sinica, Taipei 11529, Taiwan; 5 Department of Chemistry, National Taiwan University, Taipei 10617, Taiwan; 6 Core Facilities for Protein Structural Analysis, Institute of Biological Chemistry, Academia Sinica, Taipei 11529, Taiwan; 7 Institute of Plant and Microbial Biology, Academia Sinica, Taipei 11529, Taiwan; 8 Agricultural Biotechnology Center, National Chung Hsing University, Taichung 40227, Taiwan

## Abstract

Methanogens have gained much attention for their metabolic product, methane, which could be an energy substitute but also contributes to the greenhouse effect. One factor that controls methane emission, reversible protein phosphorylation, is a crucial signaling switch, and phosphoproteomics has become a powerful tool for large-scale surveying. Here, we conducted the first phosphorylation-mediated regulation study in halophilic *Methanohalophilus portucalensis* FDF1^T^, a model strain for studying stress response mechanisms in osmoadaptation. A shotgun approach and MS-based analysis identified 149 unique phosphoproteins. Among them, 26% participated in methanogenesis and osmolytes biosynthesis pathways. Of note, we uncovered that protein phosphorylation might be a crucial factor to modulate the pyrrolysine (Pyl) incorporation and Pyl-mediated methylotrophic methanogenesis. Furthermore, heterologous expression of glycine sarcosine N-methyltransferase (GSMT) mutant derivatives in the osmosensitive *Escherichia coli* MKH13 revealed that the nonphosphorylated T68A mutant resulted in increased salt tolerance. In contrast, mimic phosphorylated mutant T68D proved defective in both enzymatic activity and salinity tolerance for growth. Our study provides new insights into phosphorylation modification as a crucial role of both methanogenesis and osmoadaptation in methanoarchaea, promoting biogas production or reducing future methane emission in response to global warming and climate change.

Methane is considered to be an energy substitute for petroleum^1^,^2^, but the emission of biologically produced methane is also a critical factor in the greenhouse effect and results in extreme climate events^3^,^4^. Methanogenic archaea are exceptional in the unusual type of metabolism that they exhibit, having the ability to gain energy from reducing CO, CO_2_, formate, methanol, methylamines, or acetate as energy and carbon sources for growth^5^,^6^. Thus, methanogens have received much attention because they play a pivotal role in both the recycling of carbon compounds to useful resources and the maintenance of the global carbon flux on Earth^3^,^6^. This dual role underscores the importance of regulating both the methanogenesis process and the stress response to environmental changes for methane emission.

Methanogens can utilize three types of methanogenic pathways for energy conservation: CO_2_-reduction, aceticlastic reactions, and methyl-group reduction (methylotrophic) pathways^7^. The order *Methanosarcinales* has the widest substrate range for methanogenesis. They are widely distributed in marine and freshwater sediments, anaerobic sewage digestors, and animal gastrointestinal tracts^7^,^8^. The methanogen used in this study, *Methanohalophilus portucalensis* FDF1^T^, belongs to the *Methanosarcinales* order and is cultivated from a naturally hypersaline environment^9^. The strain FDF1^T^ utilizes only methanol, monomethylamine (MMA), dimethylamine (DMA), and trimethylamine (TMA) as carbon and energy sources for growth through the methylotrophic methanogenesis pathway. Intriguingly, a non-canonical amino acid, pyrrolysine (Pyl), can be incorporated by an in-frame amber codon (UAG) of three distinct methylamine methyltransferases, TMA methyltransferase (*mttB*), DMA methyltransferase (*mtbB*), and MMA methyltransferase (*mtmB*), to initiate methanogenesis from methylamines^10^,^11^. The utilization of Pyl is restricted to methyltransferases or other Pyl-containing proteins existing only in a few methanogenic archaea and bacteria. The importance of Pyl in methylamine-dependent methanogenesis was shown when deletion of the *amber* suppressor tRNA^Pyl^ (*pylT*) gene in *Methanosarcina acetivorans* disabled methane production from precursor MMA, DMA, or TMA^12^. Despite the functional roles of the critical components involved in the methanogenesis pathway that have been reported, there is still surprisingly little known about their regulatory networks in methanogens.

Importantly, methane produced by halophilic methanogens contributes significantly to the carbon mineralization in marine and hypersaline environments where large amounts of the greenhouse gas methane are stored^13^. Furthermore, TMA in these habitats is an important methane precursor for the methylotrophic methanogens, since TMA precursors are constantly provided by the degradation of osmolytes, like glycine betaine (betaine), for osmoregulation to cope with high external salinity^14^,^15^. Betaine is a type of quaternary ammonium compound that can equilibrate unbalanced osmolarity and is a common osmoprotectant in prokarya and eukarya^16^,^17^,^18^,^19^. The model methanogen *M. portucalensis* FDF1^T^ has been reported to possess the capability for *de novo* synthesis of betaine through three-step methylation from precursor glycine via glycine sarcosine *N*-methyltransferase (GSMT) and sarcosine dimethylglycine *N*-methyltransferase (SDMT)^20^,^21^,^22^. It is known that intracellular salt concentration regulates the expression of GSMT and SDMT in strain FDF1^T^, and the amount of monovalent ions modulates the activity of rate-limited enzyme GSMT^21^. Furthermore, drought and salt tolerance in the arabidopsis model also increased in response to heterogeneously expressed GSMT and SDMT from strain FDF1^T ^23. Despite these studies on key regulatory factors contributing to osmoadapation, there is still little evidence explaining how strain FDF1^T^ has an immediate on-off switch in the betaine synthesis process to respond to environmental changes.

Reversible protein phosphorylation is the most common cellular mechanism to regulate many physiological and adaptational processes. The first identified methanogen phosphoprotein with a known function was the methyltransferase activation protein from *Methanosarcina barkeri*, which converts methanol to methane via the methylotrophic methanogenesis pathway^24^. Furthermore, the archaeal two-component system was found to be involved in the regulation of methanogenesis in *Methanosaeta harundinacea*^25^. As genome sequences continue to accumulate, it is apparent that methanoarchaea also possess protein kinases and phosphatases, but only a few of them and their protein substrates have been defined by basic biochemical and genetic approaches^26^,^27^,^28^. Therefore, this report will be of particular use in delineating the physiological processes for methanogenesis and salt stress tolerance by displaying the global phosphorylation network from *M. portucalensis* FDF1^T^.

In this study, we provided a genome-wide, shotgun and phosphorylation site-specific investigation in a halophilic methanogen through MS-based systematic phosphoproteomic analysis. Both Ser/Thr/Tyr and His/Asp phosphorylation-based signaling systems were involved in diverse biological processes, especially in the single-carbon energy metabolism for methane production and osmolyte biosynthetic pathways. Although a previous study in the *E. coli* system had uncovered the ability to overcome hyper-salinity stress via expression of the osmolyte betaine synthesizing enzymes, GSMT and SDMT, from strain FDF1^T ^21, we further undertook a phosphosite mutagenesis analysis of GSMT (MPF_0823) to clarify its enzymatic activity and how its osmoregulatory function could be modulated by phosphorylation modification.

## Results

### Establishment of the phosphoproteome from *M. portucalensis* FDF1^T^

**Figure 1:**
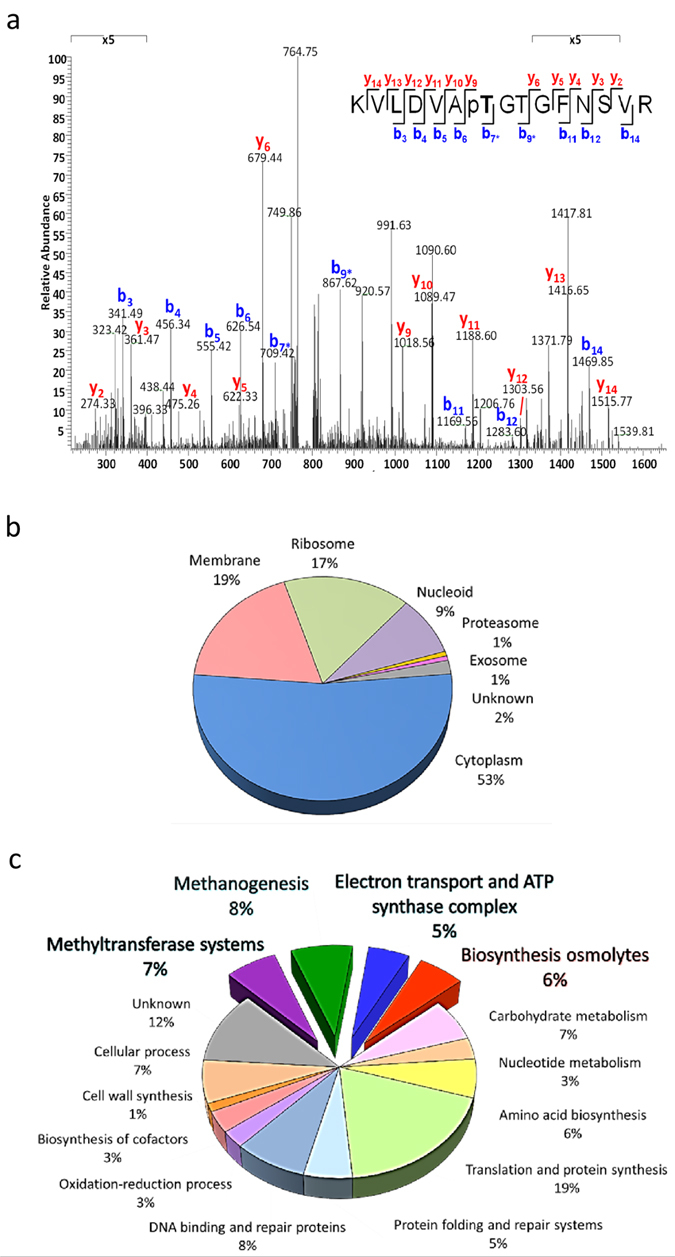
A representative MS/MS spectrum and classification of the identified phosphoproteins in *M. portucalensis* FDF1^T^. (**a**) The MS/MS spectrum acquired in the Orbitrap mass spectrometer for a threonine-phosphorylated peptide (VLDVAp*T*GTGFNSVR), in which one phosphorylated site on Thr-68 was identified from GSMT (MPF_0823). Sequence-informative fragmentation ions are summarized on the peptide sequences and the matched “y” and “b” ions are annotated in red and blue, respectively. Phosphorylation site–specific ions are indicated with “p”. Fragment ion signals corresponding to additional neutral loss of NH_3_ are indicated by asterisks. The identified phosphoproteins were grouped by (**b**) cellular locations and (**c**) biological function based on GO terms. The biological functions related to methanogenesis and osmoadaptation are highlighted as bold blue and orange, respectively. Hypothetical proteins are grouped into the “Unknown” category and the percental distribution is given.

The phosphorylation-mediated cellular signaling network in methylotrophic halophilic *M. portucalensis* FDF1^T^ was acquired through high accuracy LC-MS/MS analysis to derive a global and site-specific phosphoproteomic data set. In order to analyze a comprehensive phosphoproteome, protein extracts prepared from mid-exponential phase cultures were subjected to a combination of gel-free and gel-based approaches. In addition, TiO_2_-based HAMMOC was applied for efficient phosphopeptide enrichment^29^,^30^, and then analyzed in duplicates, as summarized in Supplementary Fig. 1. In total, we identified 308 unique phosphopeptides originating from 149 phosphoproteins with high confidence and a FDR of less than 1.0%. The distribution of class I phosphosites^31^ on Ser, Thr, Tyr, Asp, and His with a probability higher than 75.0% is listed in Supplementary Table 1. Following the typical protocols to prepare phosphopeptides, 11.8% of the phosphosites we identified were His and Asp residues. These phosphosites are thought to be mediated through two-component systems. Figure 1a exemplifies the manually annotated MS/MS spectra (full results provided in Supplementary Fig. 2), depicting the sequence VLDVAp*T*GTGFNSVR from GSMT carrying one phosphorylated site on Thr-68. Detailed information about all identified phosphopeptides and matched MS data are listed in Supplementary Table 1.

### Classification of Methanoarchaeal Phosphoproteins

The identified phosphoproteins in *M. portucalensis* FDF1^T^ were categorized by cellular localization and biological function to gain insight into their functional roles (Fig. 1b,c). Various data mining techniques using GO annotations provided useful information on the different classes, as summarized in Supplementary Table 2. Of the 149 identified phosphoproteins, 146 phosphoproteins were successfully categorized into different cellular compartments, including cytoplasm (53%), membrane (19%), ribosome (17%), nucleoid (9%), proteasome (1%), and exosome (1%) (Fig. 1b and Supplementary Table 2). As for biological function, 88.7% of the identified phosphoproteins could be assigned to 14 functional classes (Fig. 1c and Supplementary Table 1). Among them, many phosphoproteins were involved in pathways responsible for the control of key physiological processes, such as replication, transcription, translation, DNA repair systems, thermosome, proteasome, chaperone systems, osmoadaptation, and S-layer protein synthesis. To facilitate the integration of the phosphoproteome data set and uncover the functional relevance of the identified phosphoproteins, they were mapped into their complex physiological pathways as shown in Supplementary Fig. 3.

Methylotropic halophilic methanogen *M. portucalensis* FDF1^T^ can metabolize TMA via methyltransferase systems to provide electrons for reducing additional molecules to methane, thereby generating the electrochemical gradient for ATP synthesis^32^,^33^. Notably, 20% of the total identified phosphoproteins were classified in methane production for energy gain categories, such as methyltransferase systems (7%), methanogenesis (8%), and electron transport and the ATP synthase complex (5%) (Fig. 1c). Moreover, the proteins involved in osmolyte biosynthesis (6%) were associated with phosphorylation-mediated regulation, which could be essential for *M. portucalensis* FDF1^T^ to accumulate osmolytes in order to balance cell turgor under hypersaline conditions (Fig. 1c). The novel observation of these unique phosphorylation events advances our understanding of signal transduction in methanogenic archaea, especially for single carbon metabolisms in methanogenesis and osmotic adaptation, suggesting their functions in controlling numerous intracellular signaling and regulatory pathways.

### Phosphorylation in the methylotrophic methanogenesis pathway

**Figure 2:**
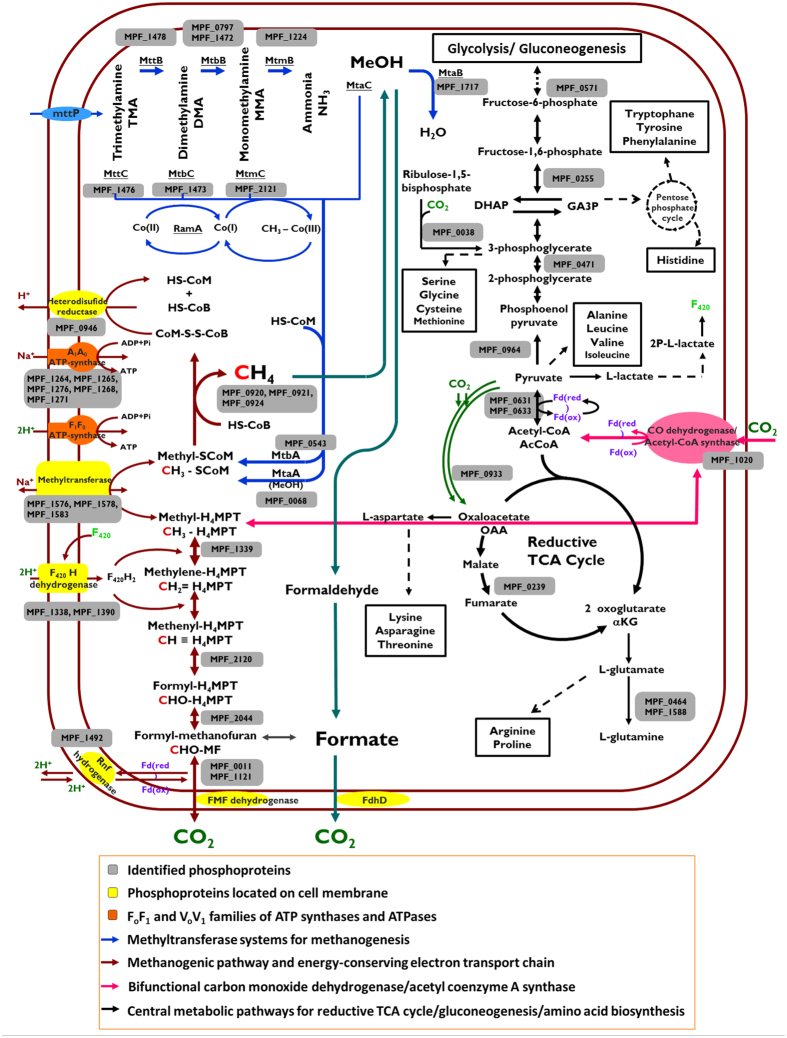
Schematic illustration of phosphorylation events in methanogenesis from TMA metabolic pathways in *M. portucalensis* FDF1^T^. Integrated phosphoproteome data mapped into methanogenic and housekeeping pathways for cells to utilize TMA, produce methane, and gain energy, as shown in different color arrows. The carbon atoms of the methanogenesis are labeled in red “C”. The shaded boxes with gene IDs (MPF_numbers) show the phosphorylated enzymes in this study.

In this study, the only carbon and nitrogen source for *M. portucalensis* FDF1^T^ growth was TMA. Ten methyltransferases initiating methylotrophic methanogenesis to convert methylamines to methyl groups were phosphorylated (Supplementary Table 1). Also phosphorylated were the proteins participating in the last methane-forming step leading up to an overall transfer of the methyl group from TMA to methane, including the methyl-CoM reductase McrAGB complex (MPF_0920, 0921, and 0924) and heterodisulfide reductase HdrD (MPF_0946). In energy-yielding processes generating a proton gradient for driving ATP synthesis, we found that five distinct ATP synthases, two membrane-bound subunits from the F_420_H_2_ dehydrogenase complex, and one electron transport RnfC could be phosphorylated (Supplementary Table 1). Likewise, we found ten phosphoproteins in carbohydrate metabolic processes such as glycolysis, gluconeogenesis, and the reductive tricarboxylic acid cycle. Figure 2 is a schematic drawing of the phosphorylation events, illustrating in greater detail these phosphoproteins participating in TMA utilization for methanogenesis and energy metabolism. These unprecedented findings suggest that protein phosphorylation globally regulates the initiation of methyl transfer reactions for the metabolism of TMA, which produces methane and generates a sufficiently positive redox potential.

### Pyrrolysine in phosphorylated methylamine methyltransferases

**Figure 3:**
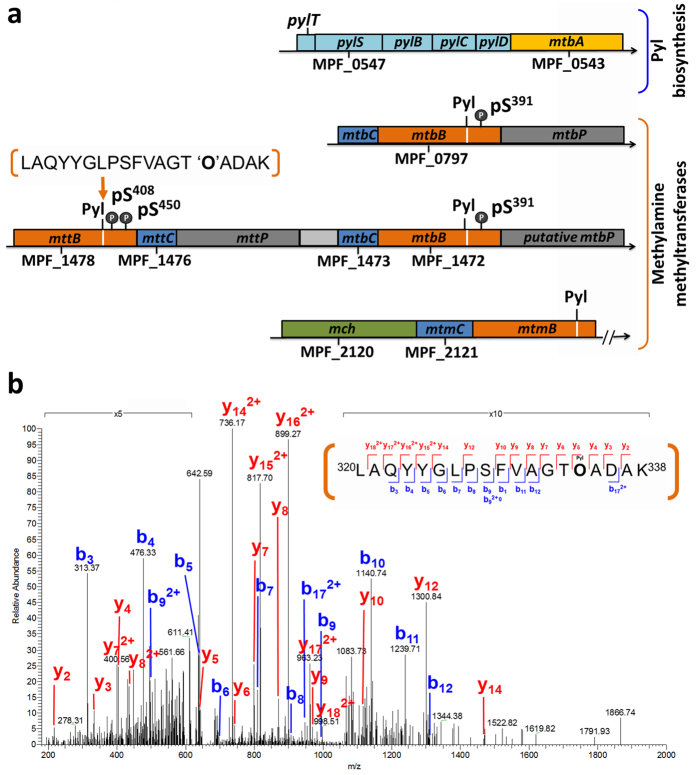
Manual annotation and MS/MS analysis reveal Pyl incorporation at amber codons and downstream phosphorylation. (**a**) The completely sequenced *M. portucalensis* genome revealed the operon (cyan) that synthesizes Pyl and incorporates it into methyltransferases. Most databases truncate methylamine methyltransferases at the amber codon, but our analysis indicated the incorporation of a Pyl residue (white line) and downstream phosphorylation on Ser-408/Ser-450 and Ser-391 in four methyltransferases (orange). (**b**) LC-MS/MS analysis confirmed the Pyl incorporation in a tryptic peptide (residues 320–338). The pyrrolysine residue “O” with a mass of 237.3 Da was detected at position 334 of TMA methyltransferase MttB (MPF_1478).

Interestingly, several phosphorylation sites were located in the C-terminal sequence downstream of the amber (UAG) codon, which encodes pyrrolysine (Pyl) during protein synthesis (Fig. 3a and Supplementary Fig. 4a). The peptide with the amber (UAG)-encoded pyrrolysyl-residue incorporated into MttB (MPF_1478) was measured by mass spectrometry as shown in Fig. 3b and Supplementary Table 4, demonstrating that Pyl can be naturally synthesized and incorporated into proteins. Furthermore, the *pylTSBCD* genes for Pyl synthesis are located within a cluster that contains the gene for methylamine-specific methyltransferase MtbA (MPF_0343) (Fig. 3a). Intriguingly, the pyrrolysyl-tRNA synthetase (MPF_0547/PylS) identified for Pyl-tRNA^Pyl^ formation included multiple phosphosites at Ser-390/Ser-391 and possibly Tyr-384/Thr-385 on the C-terminal tail of the predicted structure (Supplementary Fig. 4b), which is the core-binding surface for the tRNA^Pyl^ acceptor helix^34^,^35^,^36^. Taking into account the importance of Pyl being present in the active site of monomethylamine methyltransferase MtmB in *M. barkeri*^37^, our findings imply that the formation of the Pyl-tRNA^Pyl^ may be regulated by protein phosphorylation, thereby influencing the enzymatic integrity of methylamine methyltransferases.

### Phosphorylation in salt stress response

Unlike bacteria, most archaea lack rigid outer envelopes, like the peptidoglycan, for salt resistance^38^. They instead rely on strategies like accumulating small molecular osmolytes to overcome the high turgor pressure. *M. portucalensis* FDF1^T^ synthesizes glycine betaine *de novo* as a preferred osmolyte for turgor adjustment^21^. We found that most proteins participating in the betaine uptake and synthesis pathways were phosphorylated (Supplementary Table 1), including the glycine betaine BtaABC transporter (Supplementary Fig. 3) and enzymes involved in the methionine transmethylation cycle and betaine biosynthesis (Supplementary Fig. 5). Because GSMT was a protein known to be the rate-limiting enzyme in the production of intermediate substrate sarcosine for further betaine synthesis^21^, we investigated the phosphorylation of GSMT. Previous research had uncovered only four phosphorylated serine residues in the GSMT ortholog in rat hepatocytes, Glycine *N*-methyltransferase (GNMT)^39^. We identified one of those phosphosites in GSMT as well as nine more. The multiple phosphosites observed on GSMT included six unambiguous phosphosites with a localization probability ≥75% (Ser-46, Thr-68, Thr-70, Tyr-169, Ser-178, and Ser-179) and four putative residues scoring <75% (Ser-74, Asp-170, Asp-174, and Tyr-177) (Supplementary Table 1). Mammalian GNMT exhibits strong structural similarity with the highly phosphorylated *M. portucalensis* GSMT (MpGSMT) (Supplementary Fig. 6a), suggesting that there are more phosphorylation sites to be uncovered in GNMT, which may underscore the importance of GNMT to a further extent than the four previously identified serine residues. To better understand how cells maintain osmotic balance via protein phosphorylation, we focused on phosphorylated MpGSMT and examined the roles of the identified phosphorylation sites.

### The effects of MpGSMT phosphothreonine on methyltransferase activity and salt stress response

**Figure 4:**
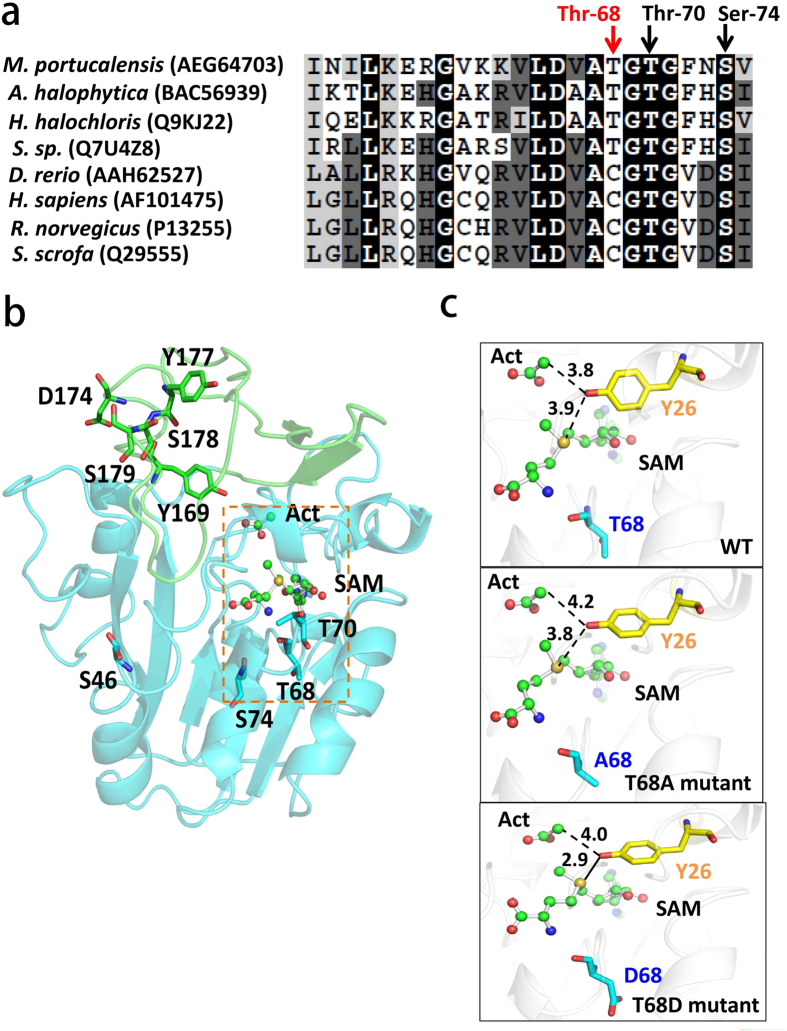
The transmethylation activity of MpGSMT was regulated via Thr phosphorylation. (**a**) Partial sequence alignment of the MpGSMT with its homologous regions from prokaryotic GSMT and eukaryotic GNMT. The amino acid sequences of GSMT were captured from *M. portucalensis* FDF1^T^, *Aphanothece halophytica*, *Halorhodospira halochloris*, and *Synechococcus* sp. WH8102, and the sequences of GNMT from *Danio rerio*, *Homo sapiens*, *Rattus norvegicus*, and *Sus scrofa*. The conserved residues are highlighted in black, while the strongly similar residues are shown in gray background. Arrows indicate the phosphorylated residue Thr-68, Thr-70, and Ser-74 located in SAM binding motif. The red arrow marks the position of phospho-residue that regulate activity in MpGSMT. (**b**) The phosphorylation sites mapped to MpGSMT predicted structure according to the rat GNMT (PDB: 1nbh) as template, which was solved with SAM and acetate mimicking glycine in catalytic pocket. The lid moiety (167–219) was shown in green ribbon and central domain containing SAM binding motif (43–166 and 220–263) was marked in blue. The phosphosites were highlighted in sticks. (**c**) An expanded view of catalytic pocket of wild-type GSMT, T68A, and T68D mutants. The Tyr-26 located near the SAM-binding site was labeled in yellow stick. The distances between Tyr-26 and SAM were labeled in dashed lines. The possible hydrogen bond was denoted in solid line with the distance of 2.9 Å.

Sequence alignment and predicted MpGSMT structure indicate that the first four phosphorylated sites (Ser-46, Thr-68, Thr-70, and Ser-74) are located in the SAM binding region, and the remaining phosphosites (Tyr-169, Asp-170, Asp-174, Tyr-177, Ser-178, and Ser-179) are located in the lid structure for substrate glycine or sarcosine binding (Fig. 4a,b). Notably, phosphosites Thr-68 and Thr-70 are situated in a highly conserved GxG motif of class I methyltransferases for the interaction with the methionine group of SAM^40^ (Fig. 4a). Intriguingly, phosphosite Thr-68 was highly conserved in prokaryotic GSMT, but the residue was replaced with a nonphosphorylatable Cys in eukaryotic GNMT, hinting at the possible regulation of MpGSMT mediated by threonine phosphorylation. Based on energy minimization of the catalytic environment of MpGSMT modeling structures (Fig. 4c), the distance between phenolic hydroxyl Tyr-26 and the sulfur atom on SAM indicate suitable geometry to form a hydrogen bond with the potential to inhibit the enzymatic reaction in the T68D phospho-mimicking mutant, but not in the other two modeling structures (WT and T68A).

**Figure 5:**
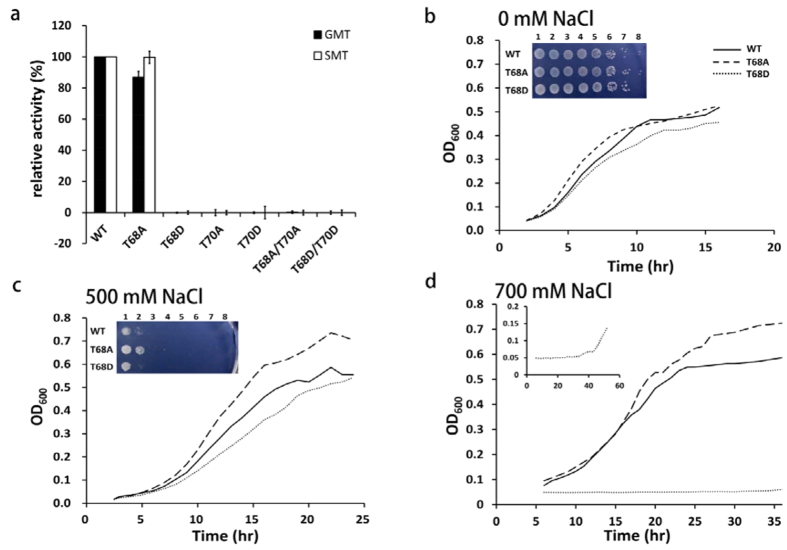
Phosphosite mutation of MpGSMT affects methyltransferase activity and growth rate at elevated osmolarity. (**a**) The relative GMT and SMT activities of recombinant WT MpGSMT and various mutant forms. The methyltransferase activities were performed by modified acid-washed charcoal method under 1.0 M of KCl with 0.5 M glycine or sarcosine as the substrate. All the data points were averaged by triplicate experiments and displayed as percentages relative to wild-type MpGSMT. The effect of salt adaptation was assayed by the growth of *E. coli* MKH13 containing WT or mutant (T68A and T68D) MpGSMT co-expressing with SDMT in M9 minimal media supplemented with (**b**) 0 mM, (**c**) 500 mM, or (**d**) 700 mM NaCl. Growth rate was monitored by measuring 600 nm optical densiometry (OD_600_) over 24 h at 37 °C. *E. coli* strain MKH13 without a recombinant GSMT^21^ had the same growth rate as the T68D mutant. The effect of salt shock shown in the inset of (**b**,**c**) was measured by drop tests of serious dilution from 10^0^ to 10^−7^ of overnight cultures on agar plates, but cells failed to grow on agar plate with (**d**) 700 mM NaCl. Insect in (**d**) indicates the growth curve of T68D mutant with a large time-scale over 48 hours.

We therefore examined the transmethylation activities by site-specific mutagenesis to determine the essential phosphorylated-threonine residues to be Thr-68 and Thr-70. While WT- and T68A-MpGSMT retained their GMT and SMT enzymatic activities, transmethylation activities were almost completely abrogated in all dephospho- (T70A and T68A/T70A) and phospho-mimetic isoforms (T68D, T70D, and T68D/T70D) (Fig. 5a). These results implied that Thr-68, but not Thr-70, could have a regulatory role for *M. portucalensis* in response to betaine biosynthesis through phosphorylation/de-phosphorylation. Additionally, circular dichroism (CD) analysis showed that the secondary structures of T68A and T68D mutant proteins had no conformational change (Supplementary Table 5), indicating that these point mutations did not affect their protein structure.

To further examine the biological effects of MpGSMT isoforms on osmoadaptation, we measured the growth of the osmosensitive strain *E. coli* MKH13 with heterologous expression of *Mpgsmt*-*sdmt* (WT) and mutant *Mpgsmt*-*sdmt* (T68A- or T68D-GSMT) genes. Media containing 0 mM, 500 mM or 700 mM NaCl were prepared in solid form for salt shock and liquid form for salt adaptation growth tests. The growth patterns of *E. coli* MKH13 with WT-, T68A- or T68D-GSMT were similar under the non-saline condition (Fig. 5b). Interestingly, *E. coli* MKH13 expressing T68A-GSMT had a fast-growth phenotype, adapting faster in 500 mM and 700 mM NaCl, while the strain expressing T68D-GSMT displayed a slow-growth phenotype (Fig. 5c,d). In other words, expression of T68A-GSMT in *E. coli* MKH13 conferred higher tolerance to salt stress than the expression of WT-GSMT (Fig. 5c,d). Conversely, the strain expressing T68D-GSMT required a longer lag period (45 hours) to overcome 700 mM NaCl salt stress (Fig. 5d). Similarly, the salt shock growth tests using solid media showed that the strain expressing T68A-GSMT possessed higher salt stress tolerance as NaCl concentration increased from 0 to 500 mM NaCl (Fig. 5c). However, none of the constructs could protect the cells against salt shock on solid media with 700 mM NaCl. Taken altogether, our results support the hypothesis that Thr-68 phosphorylation in MpGSMT could regulate the dual catalytic activities of GMT and SMT in GSMT while playing a leading role in osmoprotection.

## Discussion

Microbial methanogenesis is important in the carbon cycle, impacting climate change or contributing to renewable energy. A fair amount of research attention in methanogenic ecosystems or biosynthetic functions has therefore approached this issue^7^,^41^,^42^,^43^. In this study, we provided additional insights into the unique metabolic pathways and environmental adaptation correlated with functionally meaningful phospho-regulatory events in the halophilic methanogen *M. portucalensis* FDF1^T^. Through further investigation by site-directed mutagenesis, we clarified the role of MpGSMT phosphorylation in regulating methyltransferase activities and salt tolerance.

The 149 phosphoproteins identified in this study approximately 7.0% of all the ORFs encoded in *M. portucalensis* FDF1^T^ genome (nearly 2131 encoded proteins), compared with 2.5% of the halophilic archaeon *Halobacterium salinarum*^44^. Both archaeal phosphoprotemic analyses revealed that phosphorylated proteins were involved in almost every cellular process, though unique physiological traits such as methanogenesis and osmolyte biosynthesis were uncovered in *M. portucalensis* (Fig. 1c and Supplementary Fig. 3). We observed that many phosphoproteins were classified as translation and protein repair (Fig. 1c, Supplementary Fig. 3 and Supplementary Table 1), possibly promoting protein homeostasis to adapt to fluctuations in external osmotic pressure. This phenomenon is similarly observed in eukaryotes to buffer normal fluctuations in cellular state and maintain cellular homeostasis via phosphorylation-mediated regulation of translation or chaperone proteins^45^.

Methanogens in the *Methanosarcinaceae* family have been found in a wide variety of anaerobic environments where methane is produced from the widest substrate range; methylamines and methanol are generally used to generate energy^46^. The enzymes involved in methylotrophic methanogenesis and the coupled electron transfer were phosphorylated (Fig. 2), suggesting that methylotrophic methanogenesis might be globally regulated by protein phosphorylation. On the other hand, the formation of a multienzyme complex is known to be essential for demethylating substrates. All of the identified phosphosites from MtmC, MtbC, MttC, MtaA, and MtbA were located within the C-terminal domain with a Rossmann fold structure in C subunits or within the N-terminal segment capping the active center in A subunits (Supplementary Fig. 7), which is the interface between an A subunit and the core complex of the BC subunits^47^,^48^. Likewise, some phosphosites on MtaB, MtmB, and MttB were located in the helical layer, which surrounds the TIM barrel structure to interact with the partner C subunit and potentially the A subunit^48^. Taken together, these findings suggest that phosphorylation may influence the stability of methyltransferase quaternary structural complexes, and in turn alter their enzymatic activities in methylotrophic methanogenesis, but further biological validation is required to confirm the significance of our findings.

Notably, phosphorylated MttB was found to possess an in-frame amber codon with Pyl, and the pyrrolysyl-tRNA synthetase PylS (MPF_0547) for Pyl-tRNA^Pyl^ formation and Pyl incorporation was found to be phosphorylated (Fig. 3a and Supplementary Fig. 4). Building upon the known importance of Pyl incorporation in methanogenesis^37^,^49^, our results not only demonstrated that *M. portucalensis* FDF1^T^ is a Pyl-utilizing archaea but also suggested the possibility that protein phosphorylation plays an upstream regulatory role in facilitating methylamine metabolism.

Osmolyte biosynthesis pathways and protein refolding processes are energy consuming systems and are required to respond immediately to osmotic stresses^50^. Twelve units of ATP are required to generate one SAM, and three units of SAM are capable of synthesizing one unit of the osmolyte betaine, consuming 36 units of ATP in total^51^,^52^. From a bioenergetic point of view, phospho-regulatory mechanisms might provide a more energy-efficient strategy to maintain osmotic equilibrium by rapidly switching protein activity on or off. Results from the enzymatic activities and salinity tolerance assay in *E. coli* MKH13 (Fig. 5) demonstrated that the Thr-68-dephosphorylated MpGSMT was constitutively active in adapting to osmotic fluctuations.

Furthermore, we present the substrate-assisted catalysis model using MpGSMT_WT, T68A, and T68D mutants to illustrate that the phosphorylation-simulated mutant T68D has the potential to form a hydrogen bond between the hydroxyl group of Y26 and the sulfur atom of SAM (Fig. 4c). Because the position of residue Y26 in the predicted MpGSMT structure is a highly conserved homology of the Y21 residue of GNMT (PDB:1nbh) (Supplementary Fig. 6a), the catalytic center of both methyltransferases may be similar^53^,^54^. Accordingly, we further propose a transmethylation mechanism for the nonphosphorylated MpGSMT through the electron transferring from Tyr-26 to attack the amine group of glycine, followed by the methyl group attacking the sulfur atom of SAM, which may lead to methyl transfer from SAM to glycine, thus generating methylated glycine (sarcosine) and S-adenosylhomocysteine (SAH) (Supplementary Fig. 6b). Conversely, we speculate that phosphorylation on Thr-68 shortens the distance between Y26 and SAM (Fig. 4c) and in turn retards the transmethylation reaction, as evidenced by the T68D mutant causing an inactive form of MpGSMT (Fig. 5a). These results indicate that threonine phosphorylation negatively regulates MpGSMT for energy conservation.

It should be noted that GNMT is considered a tumor suppressor of human hepatocellular carcinoma, and the position of the phosphorylated residues in the GNMT tertiary structure is likely to affect the protein’s conformation and activity^55^. For instance, phosphosite Ser-71 of GNMT may affect the microenvironmental net charge of SAM binding pocket, and Ser-182 could modulate the tetramer switching to the dimer^39^. More surprising is the fact that phosphorylated GNMT significantly increases enzyme activity^39^, in contrast to phosphorylated MpGSMT, which presented a methyltransferase-inactive phenotype (Fig. 5a). Intriguingly, the phosphosites Ser-74 and Asp-174 identified in MpGSMT corresponding to rat GNMT Ser-71 and Ser-182 were evolutionarily conserved (Supplementary Fig. 8), while the quaternary structure of MpGSMT is regulated via potassium ion concentration but not mediated by protein phosphorylation^21^. Furthermore, the highly conserved phosphosite Thr-68 in prokaryotic GSMT was replaced by Cys-65 in eukaryotic GNMT (Fig. 4a), revealing that GNMTs in eukarya possessing constitutive activity might be due to the non-phosphorylatable residue Cys-65. It may therefore be regulated through another mechanism modulating the ratio of SAM/SAH in cells. Collectively, our recent findings revealed a tight correlation between the phospho-regulation and methyltransferase activities of MpGSMT, especially as an energy-efficient strategy in response to osmotic regulation.

In conclusion, we present the global phosphorylation-mediated cellular signaling networks in halophilic methanogen *M. portucalensis* FDF1^T^ to highlight the importance of phospho-regulation in methanogenesis and the betaine biosynthesis process. In particular, we demonstrated that the FDF1^T^ is a pyrrolysine-utilizing archaea and hypothesized that methane production via the methyltrophic methanogenesis may be regulated by protein phosphorylation in both protein translation and the methylamine metabolic process. In addition, this is the first study to elucidate the importance of Thr phosphorylation in prokaryotic GSMT, and clarifying that Thr-68 phosphorylation plays a distinct role in enzymatic activity and salt tolerance. This report advances our knowledge of the underlying mechanisms in the modulation of methanogenesis and osmotic adaptation and potentiates improved methane yields or minimized methane emission, both crucial in addressing global warming and climate change.

## Methods

### Growth conditions and protein extraction

The bacterial strains used for cloning and heterologous expression were *E. coli* DH5α and *E. coli* BL21(DE3)RIL (Novagen, Madison, WI), respectively. The archaeal strain *Methanohalophilus portucalensis* strain FDF1^T^ (=DSM 7471)^9^, was used in this study to derive the phosphoproteome.

*M. portucalensis* FDF1^T^ were cultured in medium containing 120 g L^−1^ NaCl and 20 mM trimethylamine as a sole carbon and energy source, as described in the literature 56. The harvested mid-exponential phase (OD_540_ at 0.5) cell pellets were resuspended in fresh lysis buffer (25 mM ammonium bicarbonate, PhosSTOP phosphatase inhibitor mixture tablets (Roche), 6 M urea, and 2 M thiourea) and disrupted by sonication on ice. The protein concentration was quantified by the Bradford protein assay (Bio-Rad).

### Phosphopeptide preparation and NanoLC-MS/MS Analysis

In order to compile a comprehensive phosphoproteome data set, the total protein was treated with trypsin in both gel-based and gel-free processes following procedures described in the literature^57^. Phosphopeptides from the tryptic peptides were enriched by custom-made HAMMOC tips, which were prepared using 0.5 mg TiO_2_ beads (GL Sciences, Tokyo, Japan) packed into 10-μL C_8_-StageTips, as described previously^29^,^30^.

The peptide mixtures were analyzed by online nanoflow liquid chromatography tandem mass spectrometry (LC-MS/MS) on a nanoAcquity system (Waters, Milford, MA) coupled to an LTQ-Orbitrap Velos hybrid mass spectrometer (Thermo Scientific) equipped with a PicoView nanospray interface (New Objective). Detailed detection conditions are described in Supplementary Information.

### MS/MS database search and phosphorylation site analysis

All MS and MS/MS raw data were analyzed using MaxQuant software (version 1.4.1.2) (http://www.maxquant.org/)^58^ with the built-in search engine Andromeda^59^ for phosphopeptide identification and phosphorylation site analysis. The protein sequences for the MS/MS database search consisted of an in-house draft genome sequence of *M. portucalensis* strain FDF1^T^ with all 2, 131 protein sequences and a well-annotated genome of *M. mahii* DSM 5219^32^, which shares 99.8% sequence identity with *M. portucalensis* strain FDF1^T^ and contains 1, 987 sequences (data download from the NCBI Reference Sequence database on December 3, 2013). The protein-encoding genes from the *M. portucalensis* strain FDF1^T^ genome sequence were previously predicted by Glimmer 2.13^60^, GeneMark 2.4, and GeneMark.hmm 2.1^61^ and annotated with the RefSeq Microbial Genomes database^62^ using BLASTP in standard settings (E-value < 10^−5^, identity >40%, and matched length >30%). The detailed search criteria are described in Supplementary Information. The identified phosphoproteins matched to protein sequences in *M. portucalensis* strain FDF1^T^ were reported and listed in Supplementary Table 1. The mass spectrometry proteomics data have been deposited to the ProteomeXchange Consortium^63^ via the PRIDE partner repository with the dataset identifier PXD002024. The detailed bioinformatics analyses used to further classify each identified protein are described in Supplementary Information, and all results were compiled into a data set provided in Supplementary Table 2.

### Cloning, expression, and purification of recombinant GSMT and mutant proteins

The *Mpgsmt* (GenBank: AEG64703) (EC 2.1.1.156) was cloned into the pET28a expression vector (Novagen)^21^ and then used as a template for site-directed mutagenesis^64^. Residues Thr-68 and Thr-70 of MpGSMT were substituted with Ala to mimic a non-phosphorylated state, and with Asp to mimic a phosphorylated state. The specific primers are listed in Supplementary Table 3. The pET28a-*Mpgsmt* construct and its mutant derivatives were transformed into *E. coli* BL21(DE3)RIL for heterologous expression as described in literature^21^.

### Methyltransferase activity assay

MpGSMT exhibiting GMT and SMT activities were determined in the forward direction, measuring sarcosine and dimethylglycine formation from glycine (Sigma) and sarcosine (Sigma) substrates. A modified acid-washed charcoal method was used to detect methyltransferase activities following the standard protocol described in literature^21^,^65^.

### Salt tolerance test

The plasmid pUHE21-*Mpgsmt-sdmt* co-expressing MpGSMT/SDMT was initially introduced into the osmolyte uptake mutant *E. coli* MKH13 as described in literature^21^. This plasmid was also used as a template for the point mutation of MpGSMT at residue Thr-68 to Ala or Asp for mimicking dephosphorylation and phosphorylation, respectively. The MKH13 cells harboring plasmid pUHE21-*Mpgsmt-sdmt* were defined as wild type (WT), while cells carrying the plasmid with a mutated version of *Mpgsmt* at Thr-68 were indicated as mutant T68A or T68D. For growth tests, three *E. coli* strains (WT, T68A, and T68D) were grown in M9 minimal medium without NaCl for at least three generations, and then subcultured in different NaCl concentrations in solid and liquid medium to investigate the influence of salt shock and salt adaptation, respectively. To assess the effects of salt shock, the overnight cultures of the three strains were diluted to OD_600_ of 1.0 using M9 medium without NaCl, and then used for further serial dilution to be dotted in equal volumes of each dilution, ranging from 10^0^ to 10^−7^, on M9 agar plates containing 0, 500, or 700 mM NaCl. All the conditions were carried out in triplicate and incubated at 37 °C. To assess the effects of salt adaptation, the three strains were acclimated in M9 liquid medium containing 0, 500, or 700 mM NaCl for three generations. The cells in 0 mM NaCl were inoculated at an initial OD_600_ of 0.01 for growth curves. In order to shorten the lag period prior to initiating growth in high concentrations of 500 or 700 mM NaCl, the initial OD_600_ value was fixed at 0.03. The growth rates of each strain in different salt conditions were determined in duplicate experiments.

## Additional Information

**How to cite this article**: Wu, W.-L. *et al*. Phosphoproteomic analysis of *Methanohalophilus portucalensis* FDF1^T^ identified the role of protein phosphorylation in methanogenesis and osmoregulation. *Sci. Rep.* **6**, 29013; doi: 10.1038/srep29013 (2016).

## Supplementary Material

Supplementary Information

Supplementary Table S1

Supplementary Table S2

Supplementary Table S3

Supplementary Table S4

Supplementary Table S5
