# Supplementary material for: Phosphoproteomic analysis of Methanohalophilus portucalensis FDF1T identified the role of protein phosphorylation in methanogenesis and osmoregulation
Source: Sci Rep. 2016 Jun 30;6:29013. doi: 10.1038/srep29013 (PMC4928046; doi:10.1038/srep29013)
Supplement: Supplementary Information [file srep29013-s1.pdf]

Supplementary information

Phosphoproteomic analysis of *Methanohalophilus portucalensis* FDF1^T identified the role of protein phosphorylation in methanogenesis and osmoregulation

Wan-Ling Wu[1,a], Shu-Jung Lai[1,a,b], Jhih-Tian Yang [a,h], Jeffy Chern[a,c,d], Suh-Yuen Liang[a,e], Chi-Chi Chou[a,e], Chih-Horng Kuo[f,g], Mei-Chin Lai[b,g*], and Shih-Hsiung Wu[a,c,d*]

[a] Institute of Biological Chemistry, Academia Sinica. Taipei 11529, Taiwan

[b] Department of Life Sciences, National Chung Hsing University, Taichung 40227, Taiwan

[c] Chemical Biology and Molecular Biophysics Program, Taiwan International Graduate Program, Academia Sinica, Taipei 11529, Taiwan

[d] Department of Chemistry, National Taiwan University, Taipei 10617, Taiwan

[e] Core Facilities for Protein Structural Analysis, Institute of Biological Chemistry, Academia Sinica, Taipei 11529, Taiwan

[f] Institute of Plant and Microbial Biology, Academia Sinica, Taipei 11529, Taiwan.

[g] Agricultural Biotechnology Center, National Chung Hsing University, Taichung 40227, Taiwan

[h] Ph.D program in Microbial Genomics, National Chung Hsing University and Academia Sinica, Taichung 40227, Taiwan

Supporting Materials and Methods

NanoLC-MS/MS Analysis

The tryptic peptide mixtures were loaded onto a 75-µm × 250-mm nanoACQUITY UPLC BEH130 column packed with C18 resin (Waters, Milford USA) and were separated at a flow rate of 300 nl/min using a linear gradient of 5-30% solvent B (95% acetonitrile with 0.1% formic acid) for 75 min, followed by a sharp increase to 90% B for 2 min and held at 95% B for another 11 min. Solvent A was 0.1% formic acid in water. The effluent from the HPLC column was directly electrosprayed into the mass spectrometer. The LTQ Orbitrap Velos instrument was operated in data-dependent mode to automatically switch between full scan MS and MS/MS acquisition. Instrument control was through Tune 2.6.0 and Xcalibur 2.1.

For the multistage activation (MSA)-MS/MS top20 method, full scan MS spectra (m/z 300–2000) were acquired in the Orbitrap analyzer after accumulation to a target value of 1E6 ions in the linear ion trap. Resolution in the Orbitrap system was set to R = 60,000 (all Orbitrap system resolution values are given at m/z 400). The 20 most intense peptide ions with charge states ≥2 were sequentially isolated to a target value of 5,000 and fragmented in the high-pressure linear ion trap by MSA with normalized collision energy of 35%. The neutral loss masses specified for MSA fragmentation were 97.98, 48.99 and 32.66 Da (singly, doubly and triply charged phosphopeptides). The resulting fragment ions were scanned out in the low-pressure ion trap at the normal scan rate and recorded with the secondary electron multipliers. The ion selection threshold was 500 counts for MS/MS, and the maximum allowed ion accumulation times were 500 ms for full scans and 100 ms for MSA-MS/MS measurements in the LTQ. An activation q = 0.25 and activation time of 10 ms were used. Standard mass spectrometric conditions for all experiments were: spray voltage, 2.0 kV; no sheath and auxiliary gas flow; heated capillary temperature, 200°C; predictive automatic gain control (AGC) enabled, and an S-lens RF level of 69%.

MS/MS database searching and phosphorylation site analysis

The search criteria used for phosphopeptide and phosphosite analysis were: trypsin digestion; cysteine carboxyamidomethylation (+ 57.0214 Da) as the fixed modification; methionine oxidation (+ 15.9949 Da), phosphorylation of serines, threonines, tyrosines, histidines, and aspartates, and protein N-terminal acetylation as variable modifications; up to two missed cleavage allowed; the minimum seven amino acids per peptide; and mass accuracy of 10 ppm for the parent ion and 0.6 Da for the fragment ions. False discovery rates (FDR) were estimated from the target-decoy strategy to distinguish between correct and incorrect identification. For the identification, FDR was set to 0.01 for sites, peptides and proteins. We calculated

the localization probabilities of all serine, threonine, tyrosine, histidine, and aspartate phosphorylation sites using the PTM score algorithm as previously described [1]. Phosphorylation sites were categorized into four classes based on the localization probability of phosphorylation. Class I sites had a localization probability of the phosphorylation site of at least 0.75; class II sites, between 0.75 and 0.5; class III sites, between 0.5 and 0.25; and class IV sites, below 0.25.

Bioinformatics analysis

In-house protein sequences of *M. portucalensis* FDF1^T were aligned with the reference sequences of *M. mahii* DSM 5219 from the Uniprot database (data were downloaded on October 31, 2013) using BLASTP in standard settings from stand-alone BLAST 2.2.25+ software [2] for Windows. The best matched protein ID (based on high-scoring segment pairs (HSP)), protein description, percent sequence identify, mismatched number of amino acid, and expectation value by random alignment from BLAST were reported. In addition, the protein function, catalytic activity, localization, and cross-reference to the Enzyme Code and Gene Ontology terms of the *M. portucalensis* FDF1^T proteins were obtained from the Protein Information Resource (PIR) database [3] or with bioinformatic tools, including STRAP [4], pSORTb 3.0 [5], and HMMTOP 2.0 [6] (**Supplementary Table** S**2**). To predict the putative two-component systems, the Prokaryotic 2-Component System database (http://www.p2cs.org/) [7] and the SwissRegulon website (http://www.swissregulon.unibas.ch/cgi-bin/TCS.pl) [8] were used for annotation.

Circular dichroism (CD) Spectrometry

The recombinant MpGSMT wt and its mutant proteins were analyzed by CD spectroscopy on a JASCO J-815 spectropolarimeter. CD spectra were recorded at 25°C using 0.1-cm quartz cuvettes with a wavelength range of 190–260 nm at a step size of 1.0 nm. Signal averaging time was 0.25 s and the slit bandwidth was 1 nm. All

spectra were corrected for buffer absorption. The secondary structure components were estimated by the CDPro program, fitting the CD spectra profiles with the secondary structure database by CDSSTR, SELCON3, and CONTINLL algorithms.

Phosphoprotein homology modeling

The MpGSMT (MPF_0823) homology model were obtained by the Swissmodel program (http://swissmodel.expasy.org/SWISS-MODEL.html) and the docked ligands to their homology model were generated by the MODELER software [9] with default settings in Discovery Studio 3.5 (Accelrys, San Diego, CA, USA, http://www.accelrys.com/dstudio), using an available crystal structure from the rat GNMT (PDB: 1nbh) as template. The graphical representation was performed and viewed in PyMOL (DeLano, W.L. The PyMOL Molecular Graphics System, http://www.pymol.org). We conducted molecular modeling of the remaining phosphoproteins with homologous or unknown 3D structures using the same approaches.

Supplementary Figures and Legends

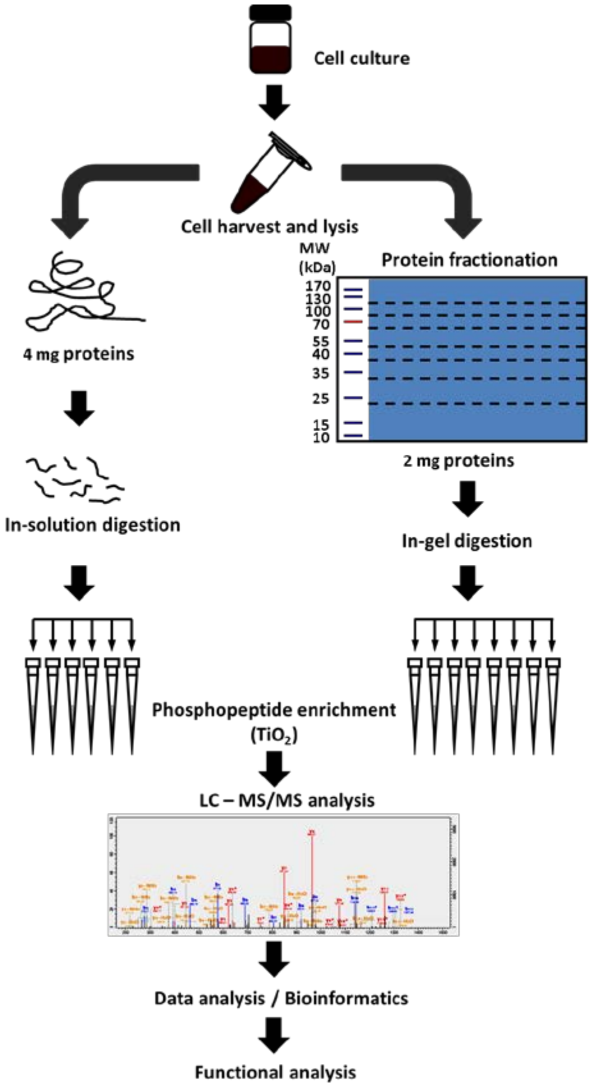

Supplementary Figure 1. Workflow for the phosphoproteomic analysis of the *M. portucalensis* FDF1^{T}

In the gel-free shotgun strategy, the harvested proteins are trypsinized and passed through six TiO_2-based HAMMOC phosphopeptide enrichment stage tips and analyzed five times on the LTQ-Orbitrap MS in duplicate. In the gel-based separation approach, the proteins electrophoresed on 12.5% SDS-PAGE were fractionated into eight fractions according to their molecular weight and then digested in-gel with trypsin for the same enrichment step followed by eight separate LC-MS runs in duplicate. Overall, 26 single injections (10 samples from in gel free method and 16 from gel-based method) were applied. All MS/MS spectra were processed by MaxQuant and searched against a target database. The identified phosphopeptides with high confidence were analyzed for data mining using bioinformatics strategies. Then, the phosphoproteins of interest from the

bioinformatics analysis were validated using functional assays.

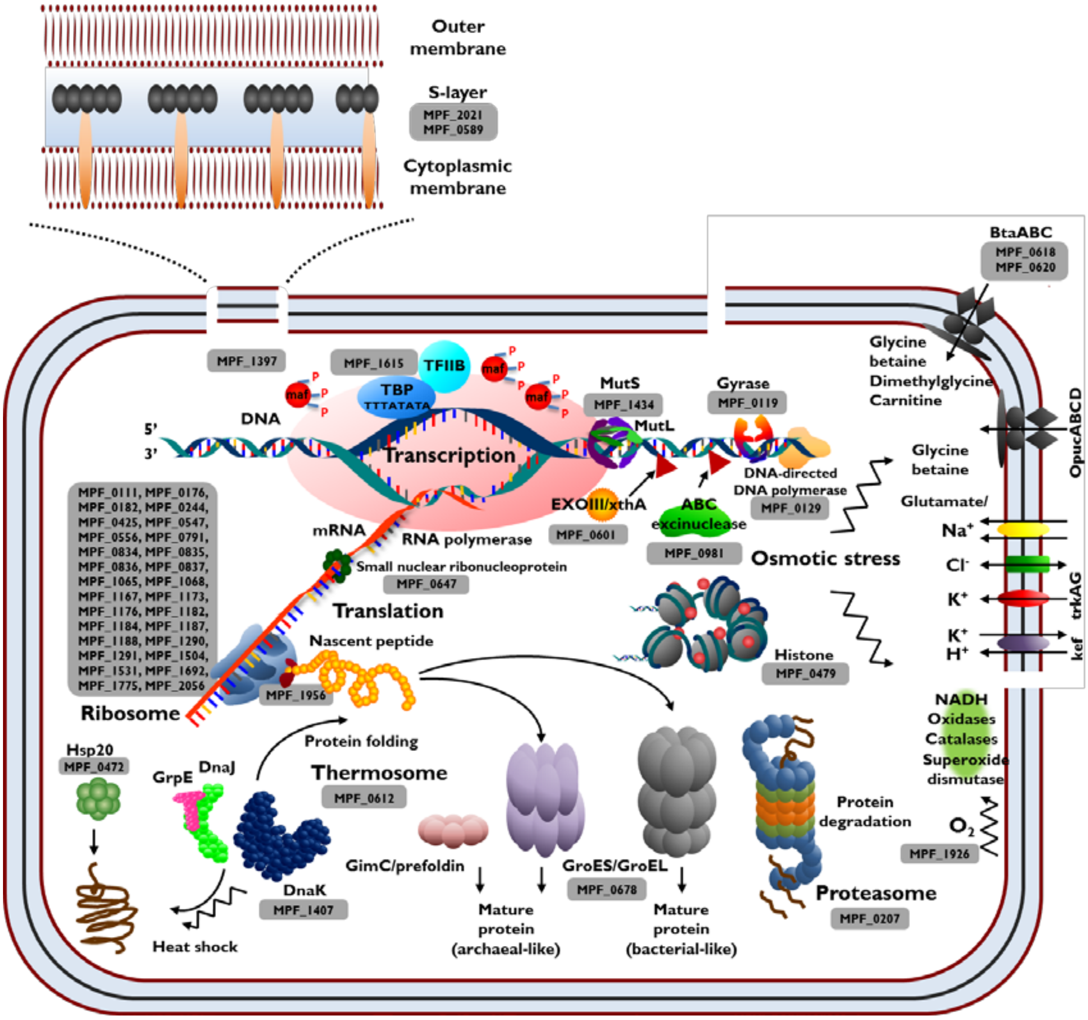

Supplementary Figure 3. Schematic of the identified phosphoproteins involved in different cellular functions of *M. portucalensis* FDF1^{T}.

The identified phosphoproteins involved in replication, transcription, translation, DNA repair systems, thermosome, proteasome, chaperone systems, osmoadaptation, and cell envelope biosynthesis are shown in shaded boxes.

(a)

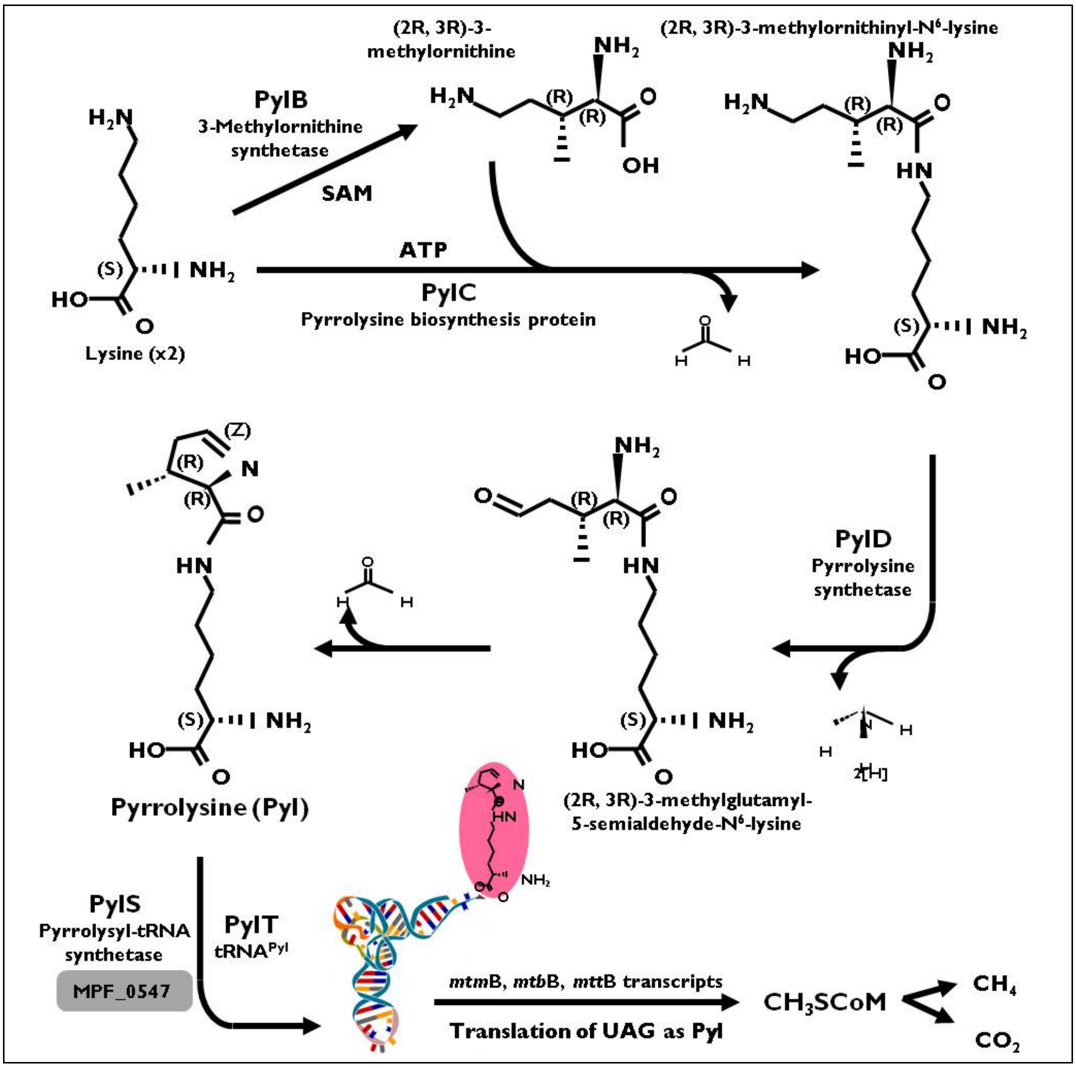

(b)

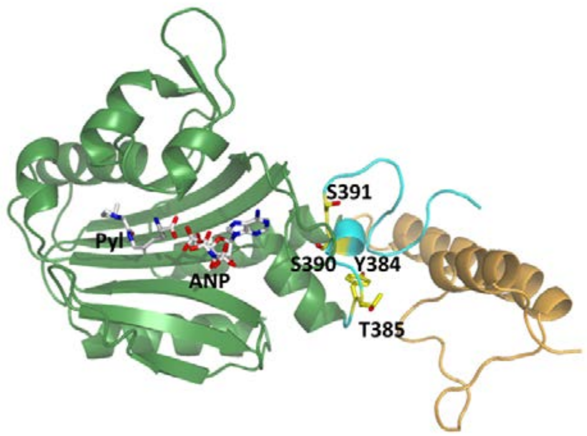

Supplementary Figure 4. Representations of pyrrolysine (Pyl) biosynthesis and incorporation into methylamine methyltransferases.

(**a**) PylB, PylC, and PylD enable Pyl production. PylS, shown in the shaded box, is a pyrrolysyl-tRNA synthetase (MPF_0547/pylS) found to be phosphorylated in this

study. PylS ligates Pyl to the $tRNA^{Pyl}$ (PylT) to form pyrrolysyl-$tRNA^{Pyl}$, and which allows Pyl to be incorporated into metabolically relevant proteins for methane production, such as the three distinct methylamine methyltransferases, MtmB, MtbB, and MttB. (**b**) Map of the identified phosphosites on the predicted PylS tertiary structures with *Methanosarcina mazei* PylS (PDB: 2E3C) as template, shown as a ribbon model. The Pyl recognition site and ATP-binding pocket are shown in green. The C-terminal tail and tRNA-binding domain 1 are blue and orange, respectively. ANP is indicated as ATP analogue. The phosphorylation sites are highlighted in yellow stick form.

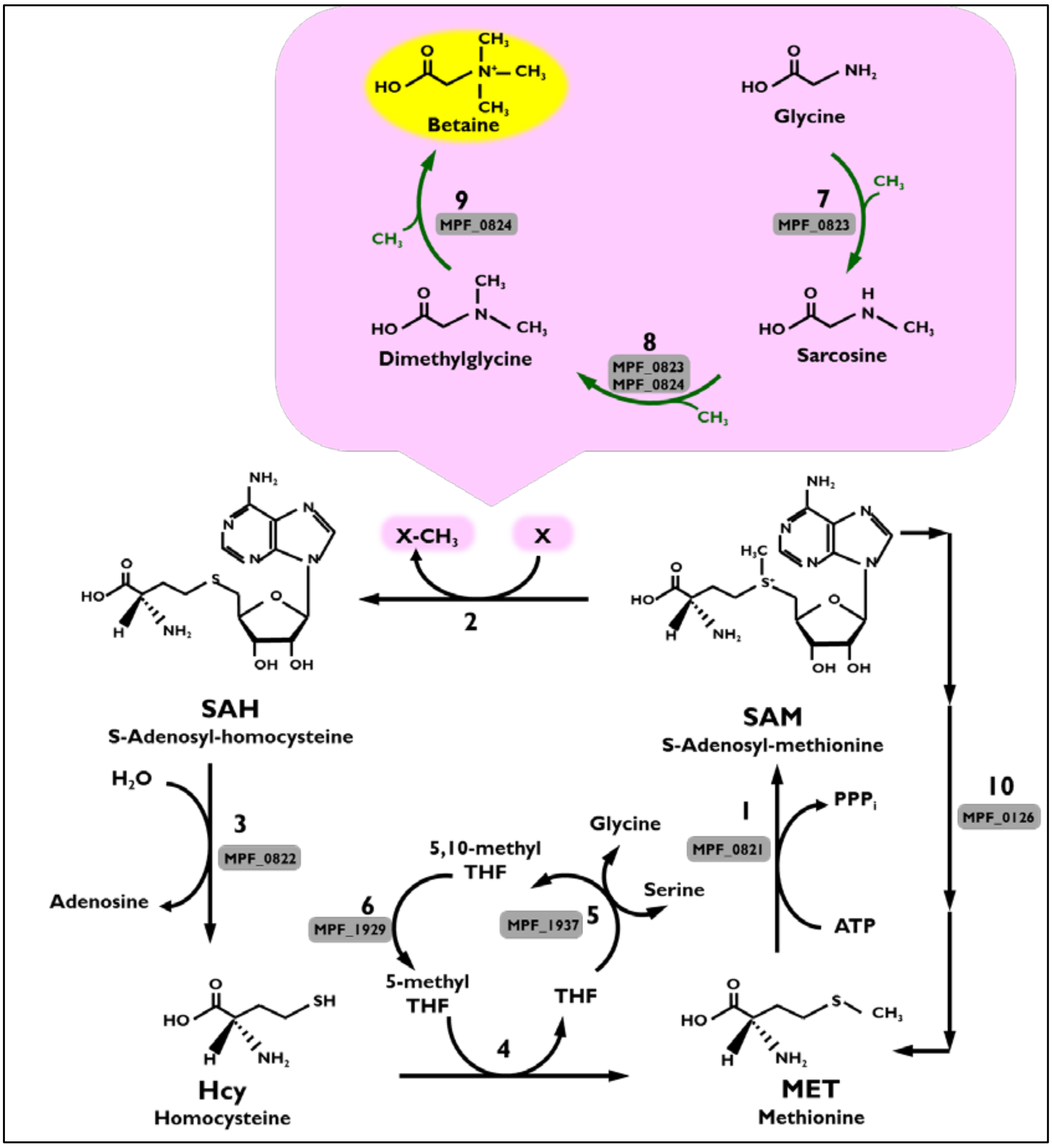

Supplementary Figure 5. Schematic of the phosphoproteins involved in the betaine biosynthetic pathway and comparison of the GSMT enzymatic activities.

The enzymes that catalyze the reactions are: 1) Methionine adenosyltransferase (MPF_0821); 2) S-adenosylmethionine-dependent methyltransferases; 3) S-adenosylhomocysteine hydrolase (MPF_0822); 4) 5-methyltetrahydrofolate homocysteine methyl-transferase; 5) Serine hydromethyltransferase (MPF_1937); 6) 5,10 methylene tetrahydrofolate reductase (MPF_1929); 7) Glycine sarcosine *N*-methyltransferase (GSMT) (MPF_0823); 8) GSMT (MPF_0823) and Sarcosine dimethylglycine *N*-methyltransferase (SDMT) (MPF_0824); 9) SDMT (MPF_0824); 10) 5-methylthioadenosine phosphorylase (MPF_0126). The two methyltransferases producing betaine with partially overlapping substrate specificity, GSMT and SDMT, are shown in the pink box. Only enzymes with

identified phosphorylation in this study are shown in the shaded boxes. Tetrahydrofolate is abbreviated as THF.

(a)

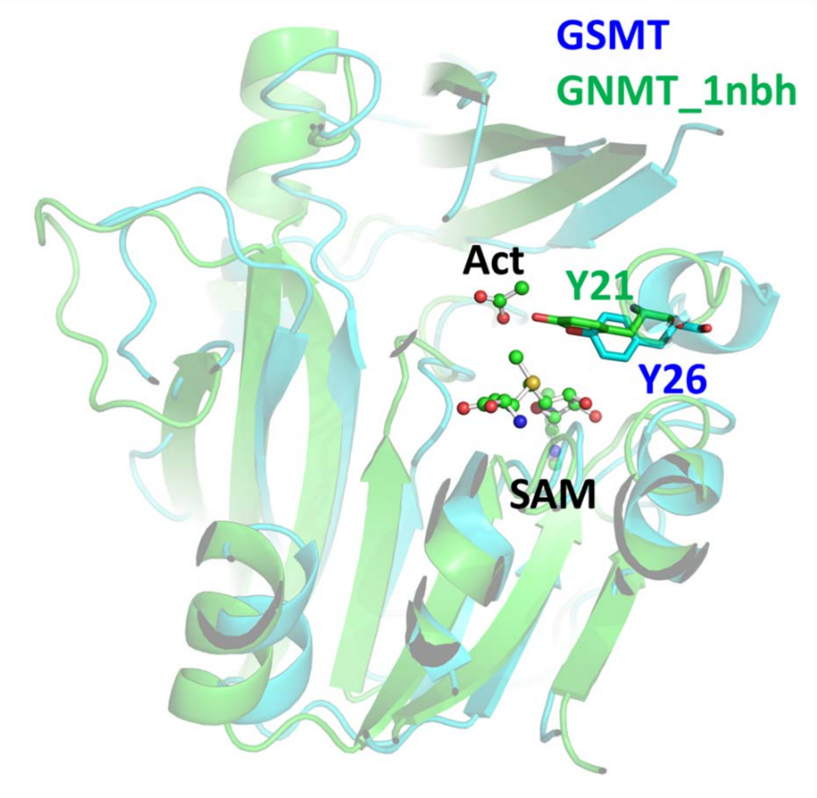

(b)

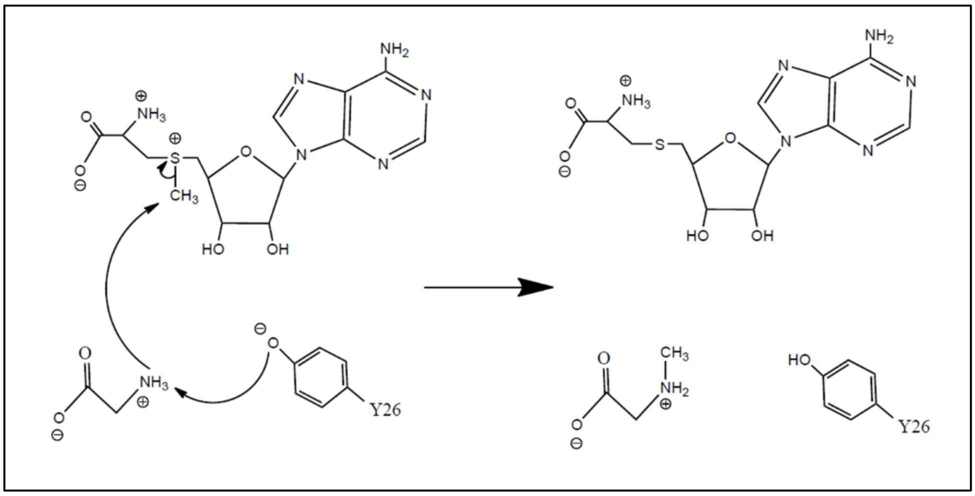

Supplementary Figure 6. Schematic of the transmethylation mechanism from prokaryotic GSMT in the nonphosphorylated state.

(a) The predicted GSMT structure (blue) was superimposed with its orthologous enzyme, GNMT (PDB:1nbh) from eukarya (green). Residue Tyr-26 (Y26) in GSMT is highly conserved as Tyr-21 (Y21) in GNMT. (b) The proposed transmethylation mechanism, with the binding of substrate glycine and SAM as the first step. The hydroxyl group on the highly conserved Y26 donates the electron to attack the amine group of glycine, and then attacks the methyl group on SAM. This reaction depolarizes the sulfur atom from SAM, leading to release of the methyl group to glycine, which produces the major end products of the methylated glycine

(sarcosine) and S-adenosylhomocysteine (SAH).

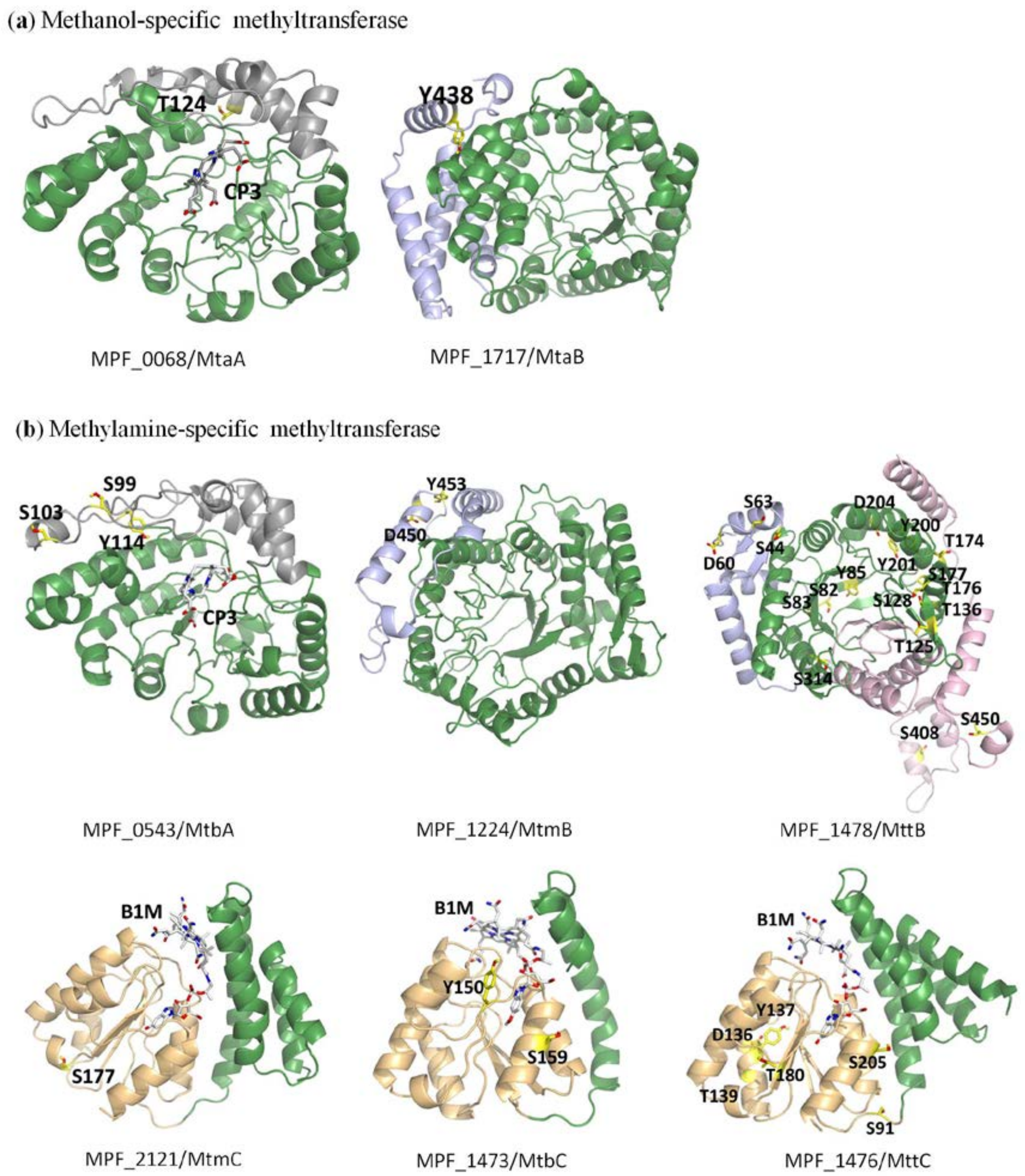

Supplementary Figure 7. Phosphorylation sites mapped to the predicted (a) methanol- and (b) methylamine- specific methyltransferase structures.

The TIM-barrel-like folds from MtaA (MPF_0068), MtbA (MPF_0543), MtaB (MPF_1717), MtmB (MPF_1224), and MttB (MPF_1478) are shown in green ribbons. The TIM-barrel-like fold in MtaA and MtbA are bound with uroporphyrinogen III (CP3). The phosphorylation sites Thr-124 from MtaA and Ser-99/Ser-103/Tyr-114 from MtbA are located within the N-terminal segment (in gray). The phosphosites Tyr-438 from MtaB, Asp-450/Tyr-453 from MtmB, and Ser-44/Asp-60/Ser-63 from MttB are located in the helical layer (in light blue), which is surrounded the TIM barrel structure (in green) for contact with the partner subunit BC complex and perhaps also subunit A. All of the phosphorylation sites from MtmC,

MtbC, and MttC were located within their Rossmann domain (in orange) for the binding position of the cob(I)alamin molecule (B1M) which is shown in stick form with nitrogen (blue) and oxygen (red). There are two phosphosites, Ser-408 and Ser-450, from MttB that are situated in the C-terminal sequence (in pink), which is downstream of the in-frame and read-through amber (UAG) codon (Figure 3). Side chains of the labeled phosphosites are represented in yellow sticks and red for oxygen. These models of full-length sequences were obtained with high confidence.

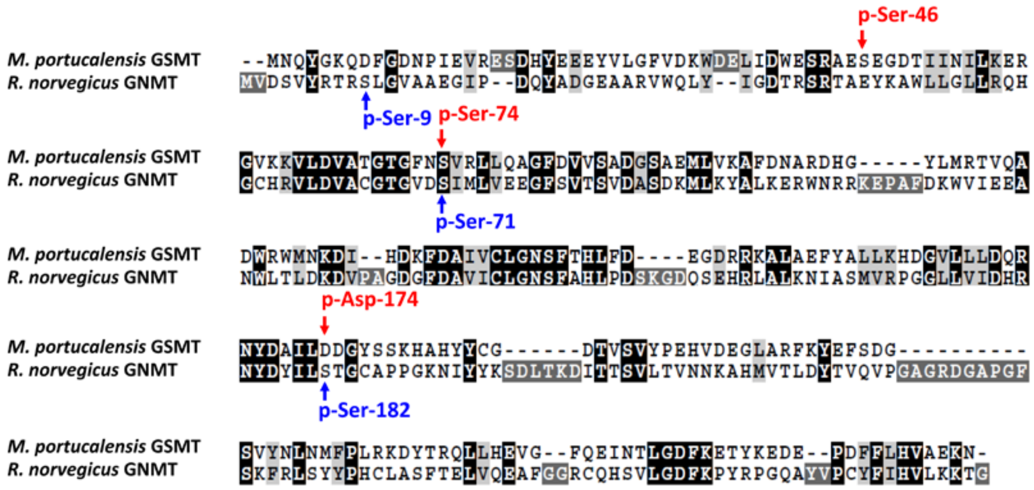

Supplementary Figure 8. Sequence alignment of *M. portucalensis* GSMT and *R. norvegicus* GNMT.

The phosphorylated residues from MpGSMT were marked with red arrows, while the corresponding sites identified in GNMT were all serine residues and marked with blue arrows. Black boxes show identity, gray boxes show similarity, and dashed lines represent gaps in MpGSMT.

Supplementary References

1. Olsen, J. V. *et al.* Global, in vivo, and site-specific phosphorylation dynamics in signaling networks. *Cell* **127**, 635-648 (2006).

2. Altschul, S. F. *et al.* Gapped BLAST and PSI-BLAST: a new generation of protein database search programs. *Nucleic Acids Res* **25**, 3389-3402 (1997).

3. Huang, H. *et al.* A comprehensive protein-centric ID mapping service for molecular data integration. *Bioinformatics* **27**, 1190-1191 (2011).

4. Bhatia, V. N., Perlman, D. H., Costello, C. E. & McComb, M. E. Software tool for researching annotations of proteins: open-source protein annotation software with data visualization. *Anal Chem* **81**, 9819-9823 (2009).

5. Yu, N. Y. *et al.* PSORTb 3.0: improved protein subcellular localization prediction with refined localization subcategories and predictive capabilities for all prokaryotes. *Bioinformatics* **26**, 1608-1615 (2010).

6. Tusnady, G. E. & Simon, I. The HMMTOP transmembrane topology prediction server. *Bioinformatics* **17**, 849-850 (2001).

7. Barakat, M., Ortet, P. & Whitworth, D. E. P2CS: a database of prokaryotic two-component systems. *Nucleic Acids Res* **39**, D771-776 (2011).

8. Burger, L. & van Nimwegen, E. Accurate prediction of protein-protein interactions from sequence alignments using a Bayesian method. *Mol Syst Biol* **4**, 165 (2008).

9. Sali, A. Comparative protein modeling by satisfaction of spatial restraints. *Mol Med Today* **1**, 270-277 (1995).
